# Editorial: Advanced approaches identifying novel nutrient-use-enhancing biostimulants

**DOI:** 10.3389/fpls.2024.1542150

**Published:** 2024-12-23

**Authors:** Hans Motte, Kris Audenaert, Lukas Spichal

**Affiliations:** ^1^ Department of Plant Biotechnology and Bioinformatics, Ghent University, Ghent, Belgium; ^2^ Center for Plant Systems Biology, VIB, Ghent, Belgium; ^3^ Laboratory of Applied Mycology and Phenomics, Department of Plants and Crops, Faculty of Bioscience Engineering, Ghent University, Ghent, Belgium; ^4^ Czech Advanced Technology and Research Institute (CATRIN), Palacky University, Olomouc, Czechia

**Keywords:** nitrogen, phosphorus, fertilization, nutrient use efficiency, biostimulants, sustainable agriculture

Agriculture today must balance high productivity with reduced environmental impact—a particularly pressing issue for phosphorus and nitrogen, two key macronutrients in fertilizers. While both nutrients are essential for plant growth, they also present challenges. Although total soil phosphorus is generally high, much of it is immobile and unavailable for plant uptake. Excessive phosphorus application can result in runoff, polluting water systems and harming ecosystems. Similarly, excessive nitrogen leaches into groundwater or enter the microbial nitrogen cycle, leading to nitrous oxide emissions—a potent greenhouse gas that exacerbates climate change. To support both productivity and sustainability, it is vital to reduce fertilizer use without compromising crop yield. This Research Topic brings together studies focused on improving nutrient use efficiency and presents a range of innovative solutions aimed at improving nutrient use efficiency.

Three studies in this Research Topic focus on innovative methods to directly improve nitrogen availability. Given the tendency of urea and ammonium to undergo rapid biochemical reactions that limit plant uptake, approaches that control nitrogen release or inhibit the downstream biochemical reactions have been shown to successfully reduce the environmental and global warming impact of nitrogen fertilizers (Lam et al., 2022).

Yan et al. introduce poly(aspartic acid)-coated urea as an environmentally friendly and cost-effective controlled-release fertilizer. Previous research has shown the efficacy of this coating in maize and rice, but Yan et al. provide new insights for wheat, demonstrating that poly(aspartic acid) effectively improves nitrogen utilization and reduces the need for frequent fertilizer application.

Nitrification inhibitors represent another important approach to slowing nitrogen loss (Beeckman et al., 2024). While most current inhibitors target ammonia-oxidizing bacteria, Beeckman et al. focus on ammonia-oxidizing archaea (AOA). Using high-throughput assays, they offer a pioneering approach to developing inhibitors specifically effective against AOA, potentially improving NUE and reducing nitrogen-related pollution. Bozal-Leorri et al. go one step further using novel wheat lines that suppress soil nitrification by releasing metabolites from roots. Interestingly, these lines inhibit nitrification in both archaea and bacteria, providing a promising strategy for sustainable nitrogen management in agriculture.

In line with the nitrogen use topic, Gao et al. explore the impact of biochar on nitrogen uptake in rice. Biochar is known to improve nitrogen efficiency by increasing nitrogen retention in soils and reducing leaching and nitrous oxide emissions (Liu et al., 2018). However, the underlying mechanisms are still unclear. Gao et al. demonstrated that biochar extracts, composed of 21 organic molecules, can enhance ammonium uptake in rice. Molecular docking studies suggest that biochar compounds may interact with a rice ammonium transporter to facilitate this effect. Hence, biochar seems to (at least partially) stimulate a plant process to improve nutrient use efficiency.

While nitrogen use is a focus, phosphate use efficiency (PUE) remains equally critical, especially given phosphorus’s limited availability in soils. PUE is known to significantly be affected by root traits (Crombez et al., 2019), which was also confirmed in cotton by Kayoumu et al., in particular for the root density. They further showed that a large plasticity in root morphological traits is important to adapt to phosphate starvation conditions, while their evaluation of 384 cotton genotypes provide a valuable basis for breeding strategies focused on nutrient-efficient genotypes. Manzoor et al. delve into the role of phosphatases—enzymes that play a crucial role in phosphorus acquisition by plants. Their work underscores the importance of interactions between root exudates and microbial phosphatase activity in enhancing phosphorus bioavailability, offering a new perspective on balancing phosphorus acquisition and restoration in soils. Finally, Magnum de Oliveira Silva et al. identify potential biochemical markers for phosphate use efficiency in eucalyptus by comparing the metabolomic and lipidomic profile of different eucalyptus species with varying phosphate use efficiency. These markers could serve as targets for future breeding programs aimed at cultivating P-efficient eucalyptus varieties.

Lastly, Chaudhary et al. investigate the potential of seaweed extracts as biostimulants in saffron production. Seaweed can be sustainably harvested along coastlines and is well-documented to have positive effects on a wide variety of plants and crops (De Saeger et al., 2020). Chaudhary et al. demonstrated that also for the economically valuable saffron, seaweed extracts improved yield.

Taken together, the studies in this Research Topic illustrate different approaches to tackle nutrient use challenges in agriculture, advancing our understanding of how biostimulants and other strategies can optimize nutrient use in agriculture. With this knowledge, future research and development can continue to deliver innovations that reduce environmental impact of agriculture while maintaining high productivity, bringing us closer to resilient and sustainable agricultural systems.
